# Osteonecrosis of the jaws in patients under osteoporosis treatment: a nine-year experience report

**DOI:** 10.20945/2359-3997000000612

**Published:** 2023-05-10

**Authors:** Daniela Cia Penoni, João Vitor S. Canellas, Marcos Antonio Nunes Costa Silami, Flávia Sader, Gonçalo Sobreira Pimentel, Anna Thereza Thomé Leão

**Affiliations:** 1 Marinha do Brasil Hospital Naval de Brasília Departamento de Saúde Brasília DF Brasil Departamento de Saúde, Divisão de Odontologia, Hospital Naval de Brasília, Marinha do Brasil, Brasília, DF, Brasil; 2 Universidade Federal do Rio de Janeiro Faculdade de Odontologia Divisão de Periodontia Rio de Janeiro RJ Brasil Departamento de Clínica Odontológica, Divisão de Periodontia, Faculdade de Odontologia, Universidade Federal do Rio de Janeiro, Rio de Janeiro, RJ, Brasil; 3 International Platform of Registered Systematic Review and Meta-analysis Protocols Delaware United States INPLASY – International Platform of Registered Systematic Review and Meta-analysis Protocols, Delaware, United States; 4 Marinha do Brasil Clínica de Estomatologia e Patologia Oral Departamento de Clínica Odontológica Rio de Janeiro RJ Brasil Departamento de Clínica Odontológica, Clínica de Estomatologia e Patologia Oral, Marinha do Brasil, Odontoclínica Central da Marinha, Rio de Janeiro, RJ, Brasil; 5 Marinha do Brasil Divisão de Periodontia Departamento de Clínica Odontológica Rio de Janeiro RJ Brasil Departamento de Clínica Odontológica, Divisão de Periodontia, Marinha do Brasil, Odontoclínica Central da Marinha, Rio de Janeiro, RJ, Brasil; 6 Marinha do Brasil, Odontoclínica Central da Marinha Divisão de Implantodontia Departamento de Clínica Odontológica Rio de Janeiro RJ Brasil Departamento de Clínica Odontológica, Divisão de Implantodontia, Marinha do Brasil, Odontoclínica Central da Marinha, Rio de Janeiro, RJ, Brasil

**Keywords:** Osteonecrosis of the jaw bisphosphonate related, antiresorptive drugs, osteoporosis, research report, clinical protocol

## Abstract

**Objective:**

This study aimed to report the experience of medication-related osteonecrosis of the jaws (MRONJ) in osteoporotic patients for nine years, and their associated initiating factors.

**Materials and methods:**

The numbers of invasive oral procedures (IOP) (tooth extraction, dental implant placement, and periodontal procedures) and removable prostheses performed from January 2012 to January 2021 were obtained from the digital records of a large public dental center. There were an estimated 6,742 procedures performed in patients under osteoporosis treatment.

**Results:**

Two cases (0.03%) of MRONJ were registered in nine years amongst patients with osteoporosis who had dental treatment at the center. From the 1,568 tooth extractions, one patient (0.06%) developed MRONJ. There was also one case from the 2,139 removable prostheses delivered (0.05%).

**Conclusions:**

The prevalence of MRONJ associated with osteoporosis treatment was very low. The protocols adopted seem to be adequate for the prevention of this complication. The findings of this study reinforce the rare frequency of MRONJ associated with dental procedures in patients submitted to the pharmacological management of osteoporosis. An integral analysis of systemic risk factors and oral preventive strategies may be considered regularly in the dental treatment of these patients.

## INTRODUCTION

Knowledge about the systemic conditions and invasive oral procedures (IOP), which could trigger the development of Medication-related osteonecrosis of the jaw (MRONJ), has been of great interest in the last years ( 1 , 2 ). There is a need for data from different geographical areas to study the condition better. Osteoporosis and its associated fragility fractures are globally common conditions, contributing significantly to morbidity, mortality, and healthcare expenses ( 3 ). Therefore, it is important to identify and treat the disease. A “call to action” has been spurred by a multitude of scientific groups worldwide to improve care and focus on osteoporosis consequences ( 4 ).

Current pharmacologic first options for osteoporosis are bisphosphonates (BP), such as alendronate, ibandronate, risedronate, and zoledronic acid, followed by RANKL inhibitor (denosumab) ( 5 ). In the osteoporosis patient population, the incidence of MRONJ was estimated at 0.001% to 0.01%, marginally higher than the incidence in the general population (<0.001%) ( 6 ). The risk of MRONJ in patients exposed to oral BPs after tooth extraction was estimated at 0.5% ( 7 ). The risk of MRONJ in procedures requiring exposure and bone manipulation, such as dental implant placement and periodontal procedures, might be comparable to the risk associated with a tooth extraction ( 8 ). This adverse effect in patients with osteoporosis exposed to antiresorptive medications has been reported as lower than 0.001%, even considering osteoporosis treatment with injectable medication, such as Denosumab semiannually up to 10 years ( 9 ). For zoledronic acid taken annually, up to 9 years, the risk has been equally low ( 10 ).

There is no evidence to suggest that patients with osteoporosis who are receiving antiresorptive medications require special treatment concerning routine dental care or changes in standard treatment practices ( 11 ). In fact, the risk might be related to a pre-existing dental infection rather than to the surgeries *per se* ( 2 ). In the oral cavity, pathologic bone loss is mainly due to infection and inflammation, commonly seen in conditions like periodontitis, peri-implantitis, and osteomyelitis/osteonecrosis ( 12 ). Dentists should be aware of the risk of MRONJ when considering invasive procedures and in case of pressure sores from ill-fitting prostheses or significant inflammation/infection ( 13 ). Comorbidities and triggering factors play a role in MRONJ in osteoporotic patients ( 14 , 15 ). The major key factor in avoiding the occurrence of MRONJ still remains the implementation of preventive measures ( 16 ). Emphasis on optimal oral hygiene and treatment of local infection has been effective in decreasing the risk of MRONJ ( 6 ). The patient should be advised about the importance of controlling inflammation and trauma in the oral cavity in order to reduce the risk of complications. Also, the patient should be taught to identify warning signs that require urgent treatment and consultation with the oral and maxillofacial surgeon, like maxillary pain, sensitive alteration in the lower lip or chin, appearance of fistulas, purulent drainage, and bone exposure ( 17 ).

This study aimed to report the 9-year experience of MRONJ and inciting events among patients exposed to antiresorptive medications for osteoporosis management in a large population assisted in a public dental center.

## MATERIALS AND METHODS

### Study design

The present study was performed at the Brazilian Naval Dental Center (OCM – *Odontoclínica Central da Marinha* ), based on digital data on the number and types of oral procedures performed between January 2012 and January 2021. The cases of MRONJ registered in this period were analyzed, regarding the types of oral procedures linked to them. This study was approved by the *Hospital Naval Marcílio Dias* Research Ethics Committees, in Rio de Janeiro, Brazil, number 16344819.9.0000.5256, and was in accordance with the ethical standards established by the Declaration of Helsinki.

### Data collection on number and type of procedures

The databases from five clinical divisions of the center were assessed: Oral and Maxillofacial Surgery (OMFS), Implant Dentistry, Periodontics, Prosthesis, and Oral Stomatology/Pathology. The numbers of the following IOP were obtained: a) OMFS: tooth extractions (excluding third molars), alveoloplasty surgical procedures and torus removal; b) Implant Dentistry: dental implant placements, and bone grafts and guided bone regenerations; c) Periodontology: crown lengthening surgeries, scaling/root planning and periodontal surgery for extensive subgingival treatment for patients with periodontitis in advanced stages. The total number of removable prostheses concluded over nine years was also obtained.

As the complications that could represent an MRONJ case have been referred to Oral Stomatology/Pathology, a search in its database was conducted in order to identify and count MRONJ cases in patients who presented osteoporosis. MRONJ case definition followed the American Association of Oral and Maxillofacial Surgeons (AAOMS) update position: patients were considered to have MRONJ if all the following characteristics were present: current or previous treatment with antiresorptive or antiangiogenic agents; exposed bone or bone that can be probed through an intraoral or extraoral fistula in the maxillofacial region that has persisted for longer than eight weeks; and no history of radiation therapy to the jaws or obvious metastatic disease to the jaws. Patients at risk for or with established MRONJ also can present with other common clinical conditions not to be confused with MRONJ. Careful evaluation was performed to avoid misdiagnosed conditions, like alveolar osteitis, sinusitis, gingivitis and periodontitis, caries, periapical pathology, odontalgia, atypical neuralgias, fibro-osseous lesions, sarcoma, chronic sclerosing osteomyelitis, and temporomandibular joint disorders. Besides that, exposed bone or sequestra can occur in patients not exposed to antiresorptive or antiangiogenic agents ( 8 ).

Prior to 2014, also according to AAOMS, patients were considered to have MRONJ if all of the following three characteristics were present: current or previous treatment with a bisphosphonate; exposed bone in the maxillofacial region that has persisted for more than 8 weeks; and no history of radiation therapy to the jaws ( 18 ).

Osteonecrosis related to antiresorptive drugs for cancer therapy was not taken into account in the present study.

### Protocols adopted previously for oral invasive procedures

Before any IOP at the dental center, irrespective at OMFS, Implantology or Periodontology, patients who present inadequate self-oral hygiene are referred to Preventive Dentistry Division, which performs plaque control, supragingival scaling, and personalized oral hygiene instruction. If patients remain with gingival bleeding, they will be monitored up to adequate oral hygiene.

The planning for rehabilitation with osseointegrated implants requires good systemic health conditions. Patients under oncology treatment, poor glycemic control are not eligible for these procedures. The control and maintenance of prostheses and peri-implant health are scheduled on a regular basis.

Antibiotics are not usually prescribed before surgical procedures, unless the patient presents a risk for infective endocarditis or complications due to systemic diseases, like diabetes, with poor glycemic control. Meanwhile, the Implantology Division has prescribed antibiotics (amoxicillin 500 mg or clindamycin 300 mg) one hour before surgical procedures and three times a day, for seven days, on forward. Steroidal anti-inflammatories (dexamethason 8 mg) are also used one hour before implant placements.

Clinics have a protocol of use of 15 mL mouth rinse of 0.12% chlorhexidine prior to IOP, keeping it used twice a day for seven days. We recommend a minimally traumatic surgical technique, removal of any bone edges, and mucosal wound closure as standard procedures.

The Informed Consent was previously signed for all the patients before treatment. Serum CTX was usually asked for surgical procedures, as well as a short-time drug holiday of 3 months, until 2015 ( 19 ). Invasive procedures were only performed when CTX was higher than 150 pg/mL. From 2015 on, neither CTX nor drug holidays have remained on OCM protocols.

### Data analysis

Data was evaluated based on the whole amount of the invasive oral procedures and removable prostheses performed at the dental center in the studied period, irrespective of the use of antiresorptive therapy.

Osteoporosis diagnosis was based on self-reporting of the result of the dual-energy x-ray absorptiometry (DXA), not on fragility fractures. DXA exams are performed at the *Hospital Naval Marcílio Dias* . An osteoporosis case is classified according to the lowest T-score: T-score ≤-2.5 SD from peak BMD at any of these sites: lumbar spine, femoral neck or total hip

For estimating the frequency of patients under antiresorptive drug use, since each patient was not evaluated individually, the present study used a database of a previous survey ( 20 ). The aim of that epidemiologic study was to describe the profile of the patients of the Brazilian Navy's Dental Center, in addition to investigating the possible association of the number of teeth and systemic risk factors. It was based on the evaluation of 1,123 questionnaires and 750 clinical examinations performed between June 2016 to June 2017. That survey has shown that advanced age and comorbidities were associated with the lower number of teeth in the studied sample. Prevention strategies were emphasized as fundamental for reaching good oral health and functional dentition at more advanced ages. In the following, epidemiologic data collection was continued for some more months, specifically in March and September 2018, and March 2019. In total, the survey reached a total of 2,908 participants. Of them, data on osteoporosis was available for 1,686 subjects; and data on osteoporosis treatment, for 1,122 subjects.

The frequency of participants who reported use or history of use of antiresorptive drugs for osteoporosis treatment was 6.5%. Based on this patient's dental center profile, it was possible to estimate the number of invasive procedures performed, as well as the number of removable prostheses delivered in the nine-year period.

## RESULTS

### Estimated number of invasive oral procedures and removable prostheses in patients under osteoporosis treatment

The numbers of IOP and removable prostheses delivered amongst 9 years are exposed in Table 1 . The total number of IOP was 70,821. A total of 4,603 IOP were estimated in patients under antiresorptive drug use. The total number of prostheses was 32,910. A total of 2,139 prostheses were estimated for patients under antiresorptive drug use.

**Table 1:** Numbers of invasive oral procedures, removable prosthesis and MRONJ in 9 years

Procedures		Total (N)	Under antiresorptive drugs *	MRONJ cases N (frequency)
Invasive oral procedures (IOP)				
OMFS				
	Tooth extraction	24,123	1,568	1 (0.06%)
	Other (than tooth extraction)	838	55	0
Implant dentistry				
	Dental implants	2,662	173	0
	Bone grafts/guided bone regenerations	2,056	133	0
Periodontology				
	Clinical crown lengthening surgery	15,562	1,011	0
	Scaling/root planning for advanced periodontitis cases	14,672	954	0
	Periodontal surgery for extensive subgingival cleaning	10,908	709	0
Total IOP		70,821	4,603	1 (0.02%)
Removable prosthesis		32,910	2,139	1 (0.05%)
Total IOP + removable prosthesis		103,731	6,742	2 (0.03%)

* Number of procedures estimated on 6.5% of patients under antiresorptive drug according to the survey performed at the dental center.

IOP: invasive oral procedures; MRONJ: medication-related osteonecrosis of the jaw; N: number; OMFS: Oral Maxillofacial Surgery.

### MRONJ experience in 9 years

The overall experience of MRONJ among patients on oral BPs submitted to dental treatment was estimated to be 0.03% (2 in 6,742). Concerning IOP, the frequency was 0.02% (1 in 4,603 IOP). Specifically, on tooth extraction, the estimated frequency was 0.06% (1 in 1,568 tooth extractions); and considering ill-fitting prosthesis, it was 0.05% (1 in 2,139 removable prostheses).

It was not identified any case of MRONJ related to procedures performed by the Implant Dentistry or Periodontology.

### MRONJ cases

Two cases of MRONJ were registered in the nine-year period ( Table 2 ). Both elderly women were 74 years old, reported more than three years of alendronate intake, and presented comorbidities and medications associated. One case was identified in the lower jaw; the other in the upper jaw.

**Table 2:** Baseline demographic and disease characteristics of the two patients who developed MRONJ

Case	Age at ONJ onset	Area	Smoking habit	Comorbidity	Medications	Cumulative dosage
1	74	Left lower jaw body	No	Hypertension, heart disease, diabetes, rheumatoid arthritis, liver cirrhosis, psychiatric	Amlodipine, Losartane, Carvedilol, Metformin, Glicazide, Prednisone, Calcitriol, Omeprazole, Azathioprine and Sertraline	Sodium Alendronate 5 years
2	74	Right upper jaw	No	Pre-diabetes	Calcium	Sodium Alendronate 3 years

One patient presented MRONJ Stage 1 (exposed and necrotic bone or fistulas that probes to the bone in patients who are asymptomatic and have no evidence of infection) and the other, Stage 2 (exposed and necrotic bone/fistulae that can be probed to the bone, associated with infection as evidenced by pain and erythema in the region of exposed bone with or without purulent drainage) ( 8 ). The first did not return and had the follow-up lost, while the other had the complete resolution and MRONJ healing in a 4-month period ( Table 3 ).

**Table 3:** Inciting event, invasive oral procedures and ONJ case details

Case	ONJ stage	Presence of local infection	Inciting event	Management	Time to healing (months)
1	1	Yes	Ill-fitting prosthesis	Local support	Lost follow-up
2	2	Yes	Tooth extraction	Sequestrectomy	4

The patient who had the tooth extracted had generalized periodontitis in advanced stages and a history of tooth loss due to this disease. This patient had used prophylactic antibiotics prescribed by the own physician before oral surgery (in April 2012) and had reported a drug holiday for 6 months from the alendronate intake.

## DISCUSSION

Based on the oral digital records of a large public dental center, the experience of MRONJ in patients under antiresorptive treatment for osteoporosis was very low in a 9-year period: 2 cases, which represents an overall frequency of 0.03% (2/6,742). This reinforces the rarity of these adverse effects when dealing with osteoporosis management. Moreover, the protocols adopted to assist patients with osteoporosis seem to be safe and adequate for MRONJ prevention.

The results of the present study are in accordance with others. A 10-years prospective study on denosumab use has revealed that the exposure-adjusted MRONJ rate was 5.2 per 10,000 person-years. Out of 1,621 patients who reported at least one invasive oral procedure and events (OPEs) – like scaling/root planning, tooth extraction, dental implants, natural tooth loss, and jaw surgery - there were 13 positively adjudicated cases of MRONJ. Overall, MRONJ incidence over seven years of denosumab use was 0.68% (11/1,621 patients) in women reporting invasive OPEs and 0.05% (1/1,970 patients) in women reporting no invasive OPEs ( 9 ). A Swedish study has shown that the prevalence of MRONJ was 0.04% among patients treated with oral BPs ( 1 ). Epidemiological evidence has shown an excess risk of hospital admission of 0.6/1,000 women with MRONJ associated with oral bisphosphonate use over five years ( 21 ).

A tooth extraction should not necessarily be postponed in patients receiving oral BPs when the aim is to control local infection ( 22 ). The only case of MRONJ related to tooth extraction performed in the OCM was in 2012 and occurred despite the use of prophylactic antibiotics and drug holiday recommended by the physician. It was stated that tooth extraction in patients receiving BP can be performed safely and predictably ( 23 ). Corroborating with our findings, a retrospective 6-year study involving 40 people who underwent tooth extraction while using osteoporosis medications, revealed no MRONJ in BP users, but two cases in denosumab users ( 24 ). Likewise, no established MRONJ cases were diagnosed during a Japanese study that involved 1,612 subjects on minodronic acid use and 1,617 subjects on raloxifene use. Suspected Stage 0 and 1 MRONJ was assessed by a structured questionnaire at baseline and at 6, 12, 18, and 24 months. The incidence of suspected Stage 0 and/or Stage 1 was 6.14 per 1,000 patient-years in the minodronic acid group and 3.38 per 1,000 patient-years in the raloxifene group (p = 0.13), and approximately 50%-60% of bone exposures that appeared during the study had disappeared at the next observation ( 25 ). A recent observational study revealed that tooth extraction was not a risk factor for developing MRONJ in patients receiving high-dose of bone agents, but that preserving teeth that require tooth extraction is a risk factor for developing this complication ( 26 ).

According to a European Task Force, tooth extraction does not automatically translate into an increased risk of developing MRONJ, as certain surgical procedures notably reduce the risk. Dental infections, rather than dental extraction or surgery *per se* , might represent a strong risk factor for MRONJ ( 13 ). A multicenter retrospective study has revealed that neither drug holidays nor preoperative administration of antibiotics significantly reduced the risk of MRONJ after tooth extraction. Meanwhile, bone loss or severe tooth mobility and an unclosed wound were all significantly associated with its development ( 22 ).

There were no cases of MRONJ caused by implant placement. A systematic review has shown that the risk of implant failure related to BP use is very low, comparable to the general population, and the functional and psychosocial benefits of such intervention should outweigh the associated risks to common medical conditions ( 27 ). A meta-analysis has shown that low-dose oral BP intake for osteoporosis treatment, in general, does not compromise implant therapy. Patients on antiresorptive use do not lose more implants or get more implant-related complications/failures than implant patients without BP intake ( 28 ). Some authors stated that the presence itself of the implant into the bone could be associated with the development of osteonecrosis, not only surgical insertion. The risk of developing MRONJ associated with the regeneration/ implant placement in patients with benign bone diseases is scarce, but it exists, especially in the posterior areas of the jaw, if the duration of treatment with BP is greater than 3 years, and if the patient is under therapy with systemic corticosteroids ( 1 , 29 ). We only use high-quality implant systems and brands. It should be noticed that patients with periodontitis and with 4 or more implants, as well as implants of certain brands and prosthetic therapy delivered by general practitioners are more likely to exhibit peri-implantitis. Peri-implant health is essential to prevent MRONJ since the surgical procedure, and the presence and persistence of peri-implant inflammation could trigger it ( 30 ).

Periodontitis was the most common initiating factor in studies concerning MRONJ ( 1 , 9 ). Periodontitis may have influenced MRONJ development following tooth extraction in the present study, but did not trigger this effect in any patients treated in Periodontology Division.

Improperly fitting dentures contributed to 1 amongst the 2 cases of MRONJ. Other authors also have reported MRONJ cases associated with improperly fitting dentures ( 9 , 11 ). According to current understandings of the pathophysiology of MRONJ, mucosal ulceration, either traumatic or in the form of an aphthous ulcer, could be its initial pathologic event. The mucosal trauma and infection would subsequently disrupt blood supply from the periosteal layer to the poorly vascularized superficial cortical bone and cause bone sequestration and possibly secondary infection ( 31 ).

The present study did not identify any case of osteonecrosis of the jaws not linked to BP use. However, reports of this disease in patients who never-used antiresorptive medication have become more frequent ( 32 ).

The adopted protocols recommend a minimally traumatic extraction technique, removal of any bone edges, and mucosal wound closure as standard procedures in patients receiving BP ( 13 , 16 ). Implantology adopts prophylactic antibiotic regimens. A recent meta-analysis has shown that administering prophylactic antibiotics reduced the risk of implant failures by 53% ( 33 ). Implantology and Periodontics Divisions have in common the fact that their surgeries have close wounds. The three Divisions have in common the control of plaque and periodontal infections previously to IOP ( 34 ).

Up to 2015, we have recommended a drug holiday from antiresorptive drugs for osteoporosis treatment of 3 months before IOP, as well as a serum CTX exam. These conducts have been banned from our protocols since there is no evidence supporting the efficacy of CTX and the short-term drug holiday in reducing the risk of MRONJ ( 6 , 22 , 35 ). A meta-analysis with 9 controlled studies revealed no significant difference in mean CTX values between patients with MRONJ and control participants (p = 0.31). A second meta-analysis with 4 studies showed no significant difference in risk of having an CTX value below 150 pg/mL for patients with MRONJ compared with control participants (p = 0.25) ( 36 ). Another recent systematic review and meta-analysis tested, as a prior outcome, the reliability of preoperative CTX levels lower than 150 pg/mL as a risk indicator for the development of MRONJ after an invasive dental procedure in patients receiving BP treatment. There were also no statistically significant differences between patients who developed MRONJ and patients who did not (p = 0.18). The quality of the evidence was acceptable ( 37 ). A consensus does not consider that resorption markers are useful to predict the onset of MRONJ; however, in selected clinical scenarios such as for patients at high risk due to exposure time (more than 4 years with antiresorptives), the use of steroids, and clinical conditions such as diabetes mellitus, smoking, and cancer, the physician and dentist may consider resorption markers, always keeping in mind that they offer no determined nor reliable prediction value ( 17 ). Important medical guidance has reported that patients with osteoporosis should be reviewed after 3 years (IV) or 5 years (oral) treatment with a bisphosphonate. Prospective and retrospective analyses report that the risk of new clinical fractures was 20%-40% higher in subjects who stopped treatment and vertebral fracture risk was approximately doubled ( 38 ).

### MRONJ prevention

MRONJ is associated with high cumulative doses of BP or denosumab. Severe consequences due to a long-term BP use might be related to advanced glycation end-products (AGEs) accumulation in bone via an increase in inflammation, reactive oxygen species (ROS) release, and bone turnover suppression. The damage appears to be offset by employing the lowest dosage of these drugs over a longer treatment time ( 39 ). However, significant accumulation of AGEs occurs with higher doses of BP, similar to those used for the treatment of Paget's disease ( 40 ). A recent review has stated that patients who have received low-dose therapy for less than 3 years and have no additional risk factors are regarded as being at low risk of development of MRONJ. On the other hand, patients scheduled to receive high-dose bisphosphonates or denosumab, those who have received low-dose bisphosphonates or denosumab previously for 3 years or more, and those with MRONJ risk factors are regarded as being at higher risk of development of MRONJ. The risk factors reported were: use of corticosteroids, chemotherapy or angiogenesis inhibitors, comorbidities (like diabetes mellitus, anemia, immunological disorders, hematological disease), smoking, and others. Although we have stated above that the use of antibiotics before tooth extraction did not make a difference in the results of the present study, the use of systemic antibiotics before and/or after the procedure should be considered, according to some authors ( 13 ), including a recently published position paper ( 16 ).

It's interesting to mention that the prevalence of MRONJ in antiresorptive medication treated patients seems to be increased by low serum Vitamin 25(OH)D ( 41 ). Adequate levels should be reached to ensure optimal bone health, especially in patients with osteoporosis ( 42 ). Although the protocols adopted do not require serum 25(OH)D, we recognize it is worthy on the approach of general and bone health.

### Adherence to osteoporosis treatment

Poor adherence to medical therapy is a widespread public health problem, associated with adverse effects on osteoporosis outcomes ( 5 ). Oral health professionals, through an effective patient/health professional communication, should encourage adherence to antiosteoporosis medication, explaining that the risk of MRONJ is extremely lower than fragility fractures. Dentists should also advise their patients on lifestyle measures to improve bone health, like ensuring adequate dietary calcium intake and vitamin D status ( 43 ). Informed consent on the risks of adverse effects of antiresorptive drugs use is advisable, but giving information may not generate fear in the patient ( 16 ).

It is necessary to recognize that this study has important limitations. One is related to the participant's self-reporting of their use of osteoporosis medication. Although the dentists were trained to achieve reliability in the answers, by asking for the name of the medication and frequency of use, it is noticed that some patients do not report the complete medical history. Second, our results were provided by estimated frequencies. The survey on the profile of the patients assisted by the dental center revealed a frequency of 6.5% of use/history of antiresorptive drugs. We also believe this frequency is underestimated because some patients “hide” the information fearing their ineligibility for IOP treatment. Then, the number of IOP performed in patients under bone medication shall be greater than 4,600. Besides, subjects under osteoporosis treatment are older, have more comorbidities, and may present more IOP than patients without osteoporosis treatment. Another limitation is that there was no data on serum Vit 25(OH)D of the patients identified with MRONJ in this study. Finally, eventually, some patients could have developed MRONJ and been potentially untreated or went to other locations for treatment, which could lead to a loss of power in the present study. This possibility, although exists, is very unlikely, since OCM is the reference for specialized treatments, and maintenance of treatments does not generate extra costs for patients.

Finally, this 9-year experience report has shown two cases of MRONJ in patients under antiresorptive drugs for osteoporosis treatment. Among the inciting events, one was caused by tooth extraction and another by a removable denture. An integral analysis of systemic risk factors, oral preventive strategies (including prosthesis and peri-implant health control) and non-traumatic procedures may be considered on a regular basis on the dental treatment of these patients.
